# High Variability of Molecular Isoforms of AMH in Follicular Fluid and Granulosa Cells From Human Small Antral Follicles

**DOI:** 10.3389/fendo.2021.617523

**Published:** 2021-03-02

**Authors:** Linn Salto Mamsen, Jane Alrø Bøtkjær, Stine Gry Kristensen, Susanne Elisabeth Pors, Janni Vikkelsø Jeppesen, Ajay Kumar, Bhanu Kalra, Erik Ernst, Claus Yding Andersen

**Affiliations:** ^1^Laboratory of Reproductive Biology, The Juliane Marie Centre for Women, Children, and Reproduction, Copenhagen University Hospital, Rigshospitalet, Copenhagen, Denmark; ^2^Institute of Reproductive and Developmental Biology, Department of Surgery and Cancer, Faculty of Medicine, Imperial College London, London, United Kingdom; ^3^Ansh Labs, LLC, Webster, TX, United States; ^4^Department of Obstetrics and Gynaecology, Regional Hospital of Randers, Randers, Denmark

**Keywords:** anti müllerian hormone, human follicular fluid (HFF), granulosa cells (GCs), endocrine function, AMH isoforms

## Abstract

Anti-Müllerian hormone (AMH) is a member of the TGF-β superfamily produced by follicular granulosa cells (GCs) in women from late gestation to the end of reproductive life. AMH is thought to inhibit aromatase (i.e., CYP19) expression and decrease the conversion of androgens to oestrogens, especially in small antral follicles before dominance is achieved. Thus, AMH acts as a gatekeeper of ovarian steroidogenesis. However, the exact function and processing of AMH has not been fully elucidated. The present study measured and determined AMH isoforms in human follicular fluid (FF) from small antral follicles and in human GCs using four ELISAs, western blot, and immunofluorescence analysis. We evaluated the presence of the following isoforms: full-length AMH precursor (proAMH), cleaved associated AMH (AMH_N,C_), N-terminal pro-region (AMH_N_), and active C-terminal (AMH_C_) AMH. A negative correlation between follicle diameter and the AMH forms was detected. Moreover, western blot analysis detected various AMH forms in both FFs and GCs, which did not match our consensus forms, suggesting an unknown proteolytic processing of AMH. The presence of these new molecular weight isoforms of AMH differs between individual follicles of identical size in the same woman. This study detected several AMH forms in FF and GCs obtained from human small antral follicles, which suggests that intrafollicular processing of AMH is complex and variable. Thus, it may be difficult to develop an antibody-based AMH assay that detects all AMH isoforms. Furthermore, the variability between follicles suggests that designing a recombinant AMH standard will be difficult.

## Introduction

Anti-Müllerian hormone (AMH) is a member of the TGF-β superfamily produced by human ovarian granulosa cells (GCs) in women from late foetal life to the end of reproductive life. In males, AMH is produced from early gestation by Sertoli cells in the testes and ensures regression of the Müllerian ducts. Circulating levels of AMH in males decrease at puberty (1–4).

Functionally, AMH is an important inhibitor of primordial follicle recruitment and reduces the sensitivity of preantral follicles to FSH-dependent selection for growth in mouse knockout studies (5, 6). In contrast, AMH enhances follicle growth both in size and numbers in cultured preantral rat follicles (7), suggesting that AMH acts as a growth factor for small growing follicles. However, the regulatory function of AMH in monoovulatory species may be different from that in polyovulatory species, since AMH does not affect the primordial follicle recruitment rate in sheep (8). In humans and primates, AMH may promote primary and secondary follicle growth *in vitro* either by enhancing recruitment and survival and/or by enhancing follicle growth; however, later in folliculogenesis, AMH seems to inhibit antral follicle formation and dominant follicle selection (9–12). Another *in vitro* study found that AMH suppressed initiation of primordial follicle growth in human ovarian tissue (13). Taken together, the mechanisms and functions of AMH in follicular recruitment and development may differ between species and may exert species-specific effects.

Another suggested function of AMH is inhibition of the aromatase (i.e., CYP19) expression leading to a decrease in the conversion of androgens to oestrogens in follicles prior to final selection. Intrafollicular AMH concentrations are especially high in small antral follicles below a diameter of 8 mm and may reach a concentration a thousand times higher than those in circulation (14). One function of AMH may act as a gatekeeper of ovarian steroidogenesis to ensure that small follicles are relatively quiescent; this would allow a direct ovarian/pituitary dialog regulating the development of the selected dominant follicle (15–19). Culturing human granulosa-lutein cells has shown that AMH suppresses the expression of both aromatase and P450 cholesterol side-chain cleavage enzyme (P450scc), another key enzyme in steroidogenesis at both the gene and protein levels, supporting the theory that AMH may inhibit steroidogenesis (17, 20).

Furthermore, polymorphisms in both the *AMH* gene and the AMH type II receptor (*AMHR2)* gene are associated with higher serum oestradiol concentrations in women, supporting the hypothesis that AMH is involved in the regulation of GC steroidogenesis (18).

**Figure 1:**
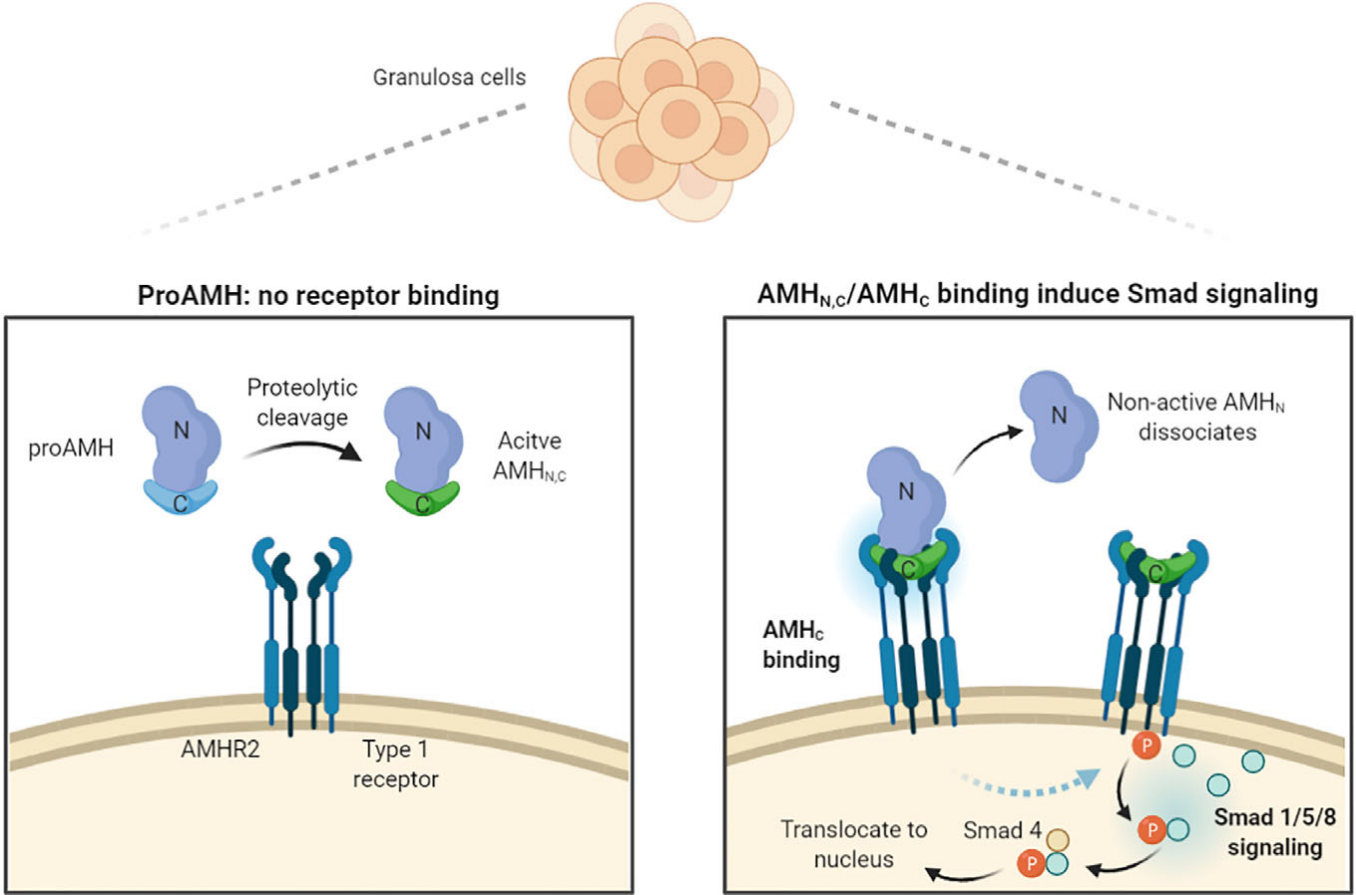
Schematic illustration of AMH processing, receptor binding, and induction of intracellular signals. AMH is produced by GCs as an inactive promature precursor (proAMH) that is not receptor competent. Proteolytic cleavage of proAMH generate a noncovalently associated form (AMH_N,C_) that can bind to the specific AMH receptor 2 (AMHR2). Upon binding to the pro region (AMH_N_) dissociates. Both AMH_N,C_ and AMH_C_ are capable of activating receptors. AMHR2 and the common Type 1 receptor phosphorylate Smads 1, 5, and 8, which bind to Smad 4, enter the nucleus and turn on AMH-responsive genes. Illustration modified from Josso (23) and created with BioRender.com.

AMH is synthesized as an inactive disulfide-like 140-kDa dimeric precursor (proAMH), comprising two 70-kDa monomers, each containing an N-terminal pro-region (AMH_N_); this is important for extracellular transport and the C-terminal (AMH_C_), which becomes bioactive upon proteolytic cleavage and undertakes receptor binding (21, 22) (**Figure 1**). The cleaved form consists of the C-terminal noncovalently associated with the N-terminal (AMH_N,C_), which is bound to AMHR2 (22, 23) (**Figure 1**). Additional proteolytic cleavage of the AMH_N,C_ complex leads to dissociation of AMH_N_ and AMH_C,_ with the AMH_C_ remaining biologically active and inducing intracellular SMAD signalling upon receptor binding (**Figure 1**) (23). ProAMH has two known cleavage sites: at amino acid (aa) 451 (between the AMH_N_ and AMH_C_ domains) and at aa 229 in the AMH_N_ region, giving rise to additional cleavage products (21, 24–26).

In contrast to other members of the TGF-β family, the N-terminal of cleaved associated AMH_N,C_ enhances the bioactivity of the C-terminal and intracellular SMAD signalling (21, 22, 27). The high concentration of AMH in FF and GCs from small antral follicles, which is almost a thousand times higher than that in circulation (19, 28), allows direct use of FF and GC in western blot analysis without any prior immunoprecipitation of AMH to avoid protein overload on the gels. This is in contrast to other previous studies that included an AMH purification step prior to western blot (27, 29). The molecular isoforms of AMH present in human FF and immature GCs have not yet been studied in detail. Moreover, it is not known whether the molecular isoforms of AMH present in FF and GCs differ between follicles within the same woman or among follicles with different diameters.

The aim of the present study was to determine the molecular isoforms of AMH present in human FFs from small antral follicles and in corresponding GCs. The study used four novel high-affinity enzyme-linked immunosorbent assays (ELISAs) to detect specific epitopes on either the AMH_C_ or the AMH_N_ form in human FF. Furthermore, FF and GC samples were analyzed using western blotting with antibodies specific to AMH_C_ and AMH_N_ and immunofluorescence analysis to determine the intrafollicular expression of AMH isoforms.

## Materials and Methods

### Patients

This retrospective study included a total of 41 female adolescents and women aged 15 to 38 years (mean age: 29.7 years) who underwent ovarian tissue cryopreservation (OTC) in 2006–2020. All patients gave informed consent to participate, and ethical approved was obtained from the Scientific Ethical Committee for the Capital Region [KF (01) 170/99].

### Human FFs and GCs

FFs and GCs were aspirated with a 1-ml syringe and 26 G needle (Becton Dickinson, Brøndby, Denmark) from follicles visible on the surface of the ovary (3–12 mm in diameter) in connection with OTC for fertility preservation (30, 31). FFs and GCs from individual follicles were handled separately, and no samples were pooled. Follicle diameter was calculated based on the aspirated volume assuming a spherical follicle (32). Follicular fluids were centrifuged at 300*g* for 2 min at room temperature to separate GCs from FF. The GCs were washed twice in PBS, and both FF and GCs were snap frozen in liquid nitrogen and stored at -80°C for further analysis.

### AMH ELISA

FFs from a total of 44 small antral follicles with a mean diameter of 6.7 mm (range: 3–11 mm) obtained from a total of 26 women were included for ELISA analysis. Four AMH ELISAs were used, each employing a specific set of antibodies that recognized different AMH sites. The AMH ELISAs used the same standardized recombinant human AMH (cat.: BA047, Ansh Labs, LLC, Webster, TX, USA) calibrators to ensure consistency between assays. ELISA analyses were performed using Ansh Labs monoclonal antibody assays against linear epitopes located on the proAMH, AMH_N,C_, AMH_N_, and AMH_C_ regions of AMH. AMH assays AL-124 (24/32) detects proAMH and AMH_N,C_ (25) (**Table 1**). Assays AL-125 (24/37) and AL-145 (10/24) detected proAMH, AMH_N,C_, and AMH_N_ (**Table 1**). AL-133 (32/33) detected proAMH, AMH_N,C,_ and AMH_C_ (**Table 1**). The known proteolytic processing of AMH [i.e., cleavage at amino acid positions 451 and 229 (20, 24, 26)] introduces conformational changes, which may change the affinity of different antibodies.

**Table 1:** AMH isoforms detected by the four different ELISA assays.

AMH forms	24/32	24/37	10/24	32/33
ProAMH	✓	✓	✓	✓
AMH_N,C_	✓	✓	✓	✓
AMH_N_	✕	✓	✓	✕
AMH_C_	✕	✕	✕	✓

### Epitope Mapping

The specificity of the AMH antibodies was investigated by assessing their binding to 80 overlapping biotinylated peptides across the precursor AMH molecule (http://www.prot.org/UniProt/P03971) as described previously (25). The peptides were synthesized by Mimotopes Pty Ltd. (Clayton, Victoria, Australia), each 12 aa long with an overlap of 4 aa; a detailed description has previously been published (33).

### Tissue Processing and Immunofluorescence Staining

One 5 mm antral follicle located in the medulla tissue was included for immunofluorescence analyses. The follicle was isolated and fixed in 10% neutral buffered formalin for 24 h, embedded in paraffin, cut into 5-µm serial sections, and processed for immunofluorescence staining. Sections were deparaffinated in xylene and rehydrated in ethanol followed by antigen retrieval in citrate buffer (10 mM sodium citrate, pH 6). Nonspecific binding was inhibited with 1% bovine serum albumin (BSA) (Sigma Aldrich, Copenhagen, Denmark). Sections were incubated with primary antibodies overnight at 4°C. Primary monoclonal mouse antibodies were specific against AMH_C_ and AMH_N_ (1:100, AMH-39/29 and 1:100, AMH-39/48, respectively, Ansh Labs, LLC, Webster, TX, USA). Sections were washed three times for 10 min in TBS with Tween 20^®^ before incubation with goat anti-mouse IgG Alexa 488 secondary antibody (1:1,000, cat.: A11029, Invitrogen, Taastrup, Denmark). Both universal negative control serum^®^ (BioCare Medical, CA, USA) and antibody dilution buffer were used in place of primary antibody as negative controls and showed no staining; data not shown. The monoclonal antibodies were screened on AMH_C_ (BA-048, Ansh Labs, LLC, Webster, TX, USA), AMH_N_ (BA-049, Ansh Labs, LLC, Webster, TX, USA), AMH_N,C_ complex (BA-047, Ansh Labs, LLC, Webster, TX, USA), and rat AMH (BA053, Ansh Labs, LLC, Webster, TX, USA) as previously described (25).

### Western Blot

Western blot analyses were performed according to the manufacturer’s instructions (Invitrogen provided by Thermo Fischer, Hvidovre, Denmark). FF samples were diluted 1:1 in nuclease-free water (cat.: AM9906, Thermo Fisher, Hvidovre, Denmark), and each lane was loaded with 1 µl of FF. GC samples from individual follicles were lysed in 20 µl of radioimmunoprecipitation assay (RIPA) buffer (cat.: R0278, Sigma-Aldrich, Brøndby, Denmark) prior to analysis. GCs from one follicle were used for up to four lanes. Samples were prepared with NuPAGE LDS 4X Sample Buffer (cat.: NP0008, Thermo Fisher, Hvidovre, Denmark) and dH_2_O. Dithiothreitol (DTT) was used as a reducing agent (cat.: NP0009, Thermo Fisher, Hvidovre, Denmark). Both reduced and nonreduced samples were heated at 95°C for 5 min before proteins were separated on a NuPAGE^®^ 4–12% Bis-Tris mini gel using NuPAGE^®^ MES SDS running buffer (cat.: NP0002, Thermo Fisher, Hvidovre, Denmark) A SeeBlue^®^ Plus2 prestained standard marker was used (cat.: LC5925, Thermo Fisher, Hvidovre, Denmark) with the following determined proteins: myosin (188 kDa), phosphorylase (98 kDa), BSA (62 kDa), glutamic dehydrogenase (49 kDa), alcohol dehydrogenase (38 kDa), carbonic anhydrase (28 kDa), myoglobin red (17 kDa), lysozyme (14 kDa), and aprotinin (6 kDa). The 188 kDa band hardly transferred to the membrane. The proteins were subsequently blotted to a PVDF membrane (Thermo Fisher, Hvidovre, Denmark). The membrane was blocked in 5% skim milk and incubated with primary antibody (AMH-39/29, 1:5,000; AMH-39/48, 1:5,000, Ansh Labs, LLC, Webster, TX, USA) overnight at 4°C and subsequently incubated with secondary horseradish peroxidase-conjugated goat-anti-mouse antibody (1:5,000, Sigma-Aldrich) for 1 hour at room temperature. Signals were detected with Pierce™ SuperSignal West Femto Substrate (Thermo Fisher) and visualized with the DNR MicroChemi 4.2 bioimaging system.

The specificity of the AMH antibodies used for western blotting has been tested (33). Surplus recombinant AMH administered together with the primary antibody resulted in the disappearance of all AMH-related bands (33). A recombinant AMH standard was used as a positive control (cat.: BA047, Ansh Labs, LLC, Webster, TX, USA).

### Statistical Analysis

Statistical analysis was performed using GraphPad Prism 8.0.0 (GraphPad Software, Inc., CA, USA). A linear regression model was used to determine associations between follicle diameter concentrations of AMH forms. Observations with missing values were excluded from the regression analysis. Significance was defined as p < 0.05.

## Results

### Levels of proAMH, AMH_N,C_, and AMH_N_ Correlated Negatively With Follicle Size

**Figure 2:**
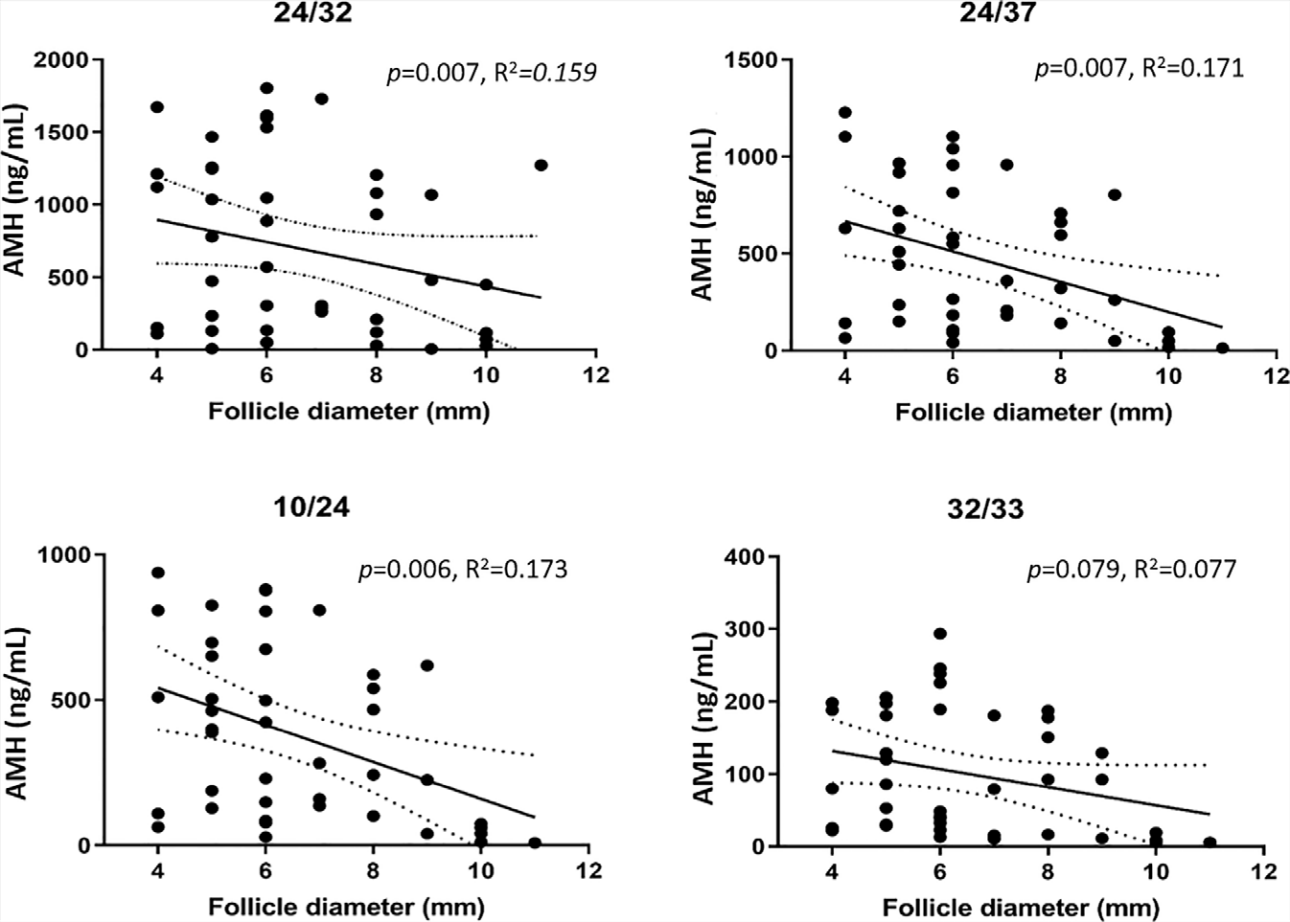
AMH concentrations measured with four different ELISAs in FFs from small antral follicles in relation to follicle size (4-11 mm). A significant negative correlation between AMH concentration and follicle size was detected with assays 24/32, 24/37, and 10/24 (Ansh Labs, LLC, Webster, TX, USA). The solid line indicates the mean AMH concentrations in relation to follicle diameter. Dotted lines indicate 95% confidence intervals.

A significant negative correlation between follicle diameter and AMH was detected with assays 24/32 (*p* = 0.007, R^2^ = 0.159), 24/37 (*p* = 0.007, R^2^ = 0.171), and 10/24 (*p* = 0.006, R^2^ = 0.1735), whereas no correlation was detected with assay 32/33 (*p* = 0.079, R^2^ = 0.077). Assay 24/32 detected proAMH and AMH_N,C_, whereas assays 24/37 and 10/24 detected proAMH, AMH_N,C_ and AMH_N_ (**Figure 2** and **Table 1**). Assay 32/33 detected proAMH, AMH_N,C,_ and AMH_C_ and is the only assay that captured and detected on the C-terminal. The AMH concentrations measured with the four different assays are presented in **Supplementary Table 1**, together with means, range and SD.

### Increased Expression of AMH_N_ and AMH_C_ in Cumulus Cells Surrounding the Oocyte

**Figure 3:**
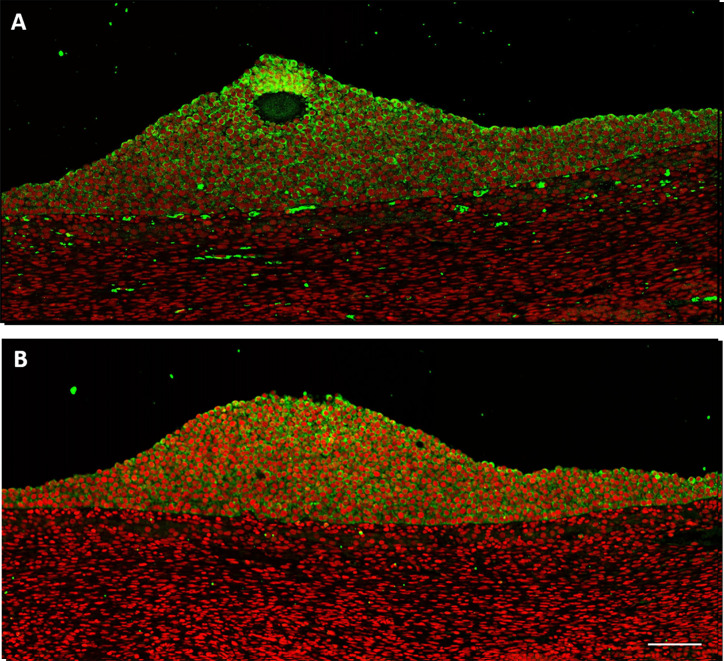
Human small antral follicle (5 mm). **(A)** AMH_C_ (green) is expressed in cumulus cells with increasing intensity toward the oocyte. Nucleus (red). **(B)** AMH_N_ (green) is expressed in cumulus cells close to the oocyte. Scale bar: 100 µm.

The cumulus cells cytoplasm in a small antral follicle (5 mm in diameter) expressed both AMH_C_ and AMH_N_ (green) (**Figures 3A, B**). The intensity of the staining increased toward the oocyte (**Figure 3A**). Autofluorescence was detected in blood vessels (**Figure 3A**) (34). Furthermore, a faint signal was detected in the theca interna. Nuclear staining was given pseudocolour red (**Figures 3A, B**).

### AMH Cleavage Products in FFs Did Not Change With Increasing Follicle Size

**Figure 4:**
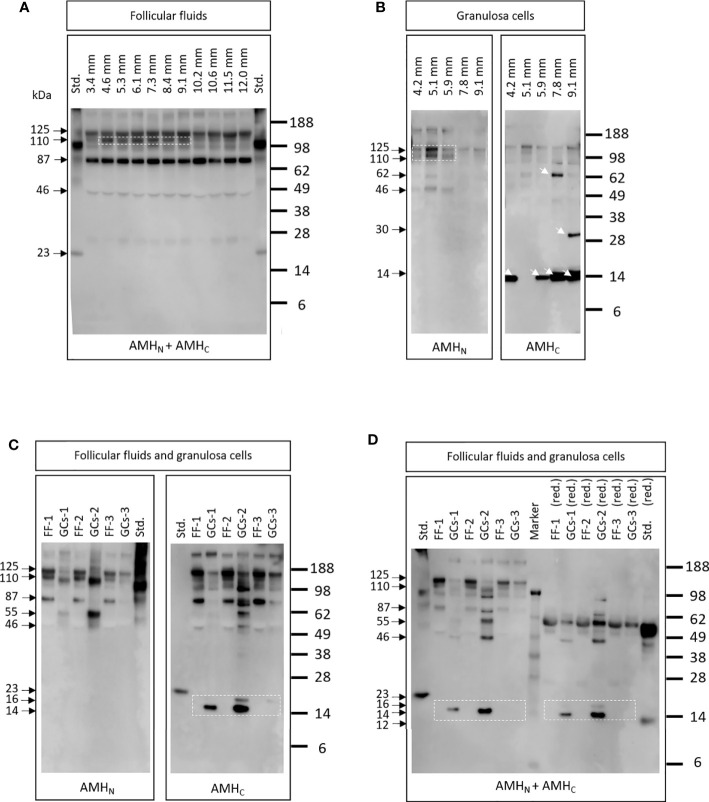
**(A)** AMH cleavage products detected in FFs obtained with increasing diameter (3.4 to 12.0 mm) from different women. Antibodies specific for epitopes on AMH_N_ and AMH_C_ were used (i.e., AMH-39/48 and AMH-39/29, respectively). A similar expression pattern was detected irrespective of follicle size, although the 110 kDa band was more pronounced in FFs from 4.6 to 9.1 mm follicles (white dotted box). Std.: 10 ng recombinant AMH standard (Ansh Labs, LLC, Webster, TX, USA). **(B)** AMH cleavage products in GCs from follicles with increasing diameter obtained from different women. Different AMH cleavage products were detected in the GCs of different follicles (white arrows). The dotted box highlights a greater expression of high molecular weight bands in GCs from the smallest follicles (i.e., 4.2-5.9 mm). Lanes are marked with the diameter of follicles where the GCs were obtained. **(C)** AMH cleavage products detected in FF and in GCs from the same follicles. Antibodies specific to AMH_N_ and AMH_C_ were used separately (i.e., AMH-39/48 and AMH-39/29). FF-1 and GG-1 are from the same follicle etc. FF-1 and FF-2 were from the same women, illustrating that different AMH forms were detected in FF and GCs from the same follicle. Note that the AMH_C_-specific antibody detected low molecular weight bands at 16 and 14 kDa in some GCs, which is not seen in FF (dotted box). Std.: 10 ng recombinant AMH standard (Ansh Labs, LLC, Webster, TX, USA). **(D)** Samples were the same as in **(C)**. Antibodies specific to AMH_N_ and AMH_C_ were used together (i.e., AMH-39/48 and AMH-39/29). Samples marked (red.) indicate that samples were treated with dithiothreitol. Low Mw bands of 16 and 14 kDa were only detected in GCs (dotted boxes) and were different from the recombinant AMH standard band at 23 kDa, suggesting that recombinant AMH does not reflect the forms present *in vivo*.

AMH cleavage products were detected using western blot analysis in FF obtained from follicles of increasing size (3.4 to 12.0 mm in diameter) from different women (**Figure 4A**). Antibodies specific for epitopes on AMH_N_ (i.e., AMH-39/48, Ansh Labs, LLC, Webster, TX, USA) and AMH_C_ (i.e., AMH-39/29, Ansh Labs, LLC, Webster, TX, USA) were used alone and together. A similar expression pattern was seen irrespective of follicle size, with bands detected at 125, 110, 87, and 46 kDa in nonreducing conditions. The 110 kDa band was more pronounced in FFs from 4.6 to 9.1 mm follicles (**Figure 4A**, dotted box). The recombinant AMH standard was detected at 23 and 100 kDa, which were different from the bands detected in FFs.

### Different AMH Cleavage Products Were Detected in GCs Obtained From Follicles With Increasing Size

AMH bands of different molecular weights were detected in GCs obtained from follicles with increasing diameters from different women (**Figure 4B**). Greater expression of high molecular weight bands was found in GCs from the smallest follicles (i.e., 4.2–5.9 mm) detected with the AMH_N_ recognizing antibody AMH-39/48 (**Figure 4B**, dotted box). Furthermore, when using the antibody specific to AMH_C,_ different cleavage products were detected in GCs obtained from follicles of increasing size (4B, arrows) during nonreducing conditions. Antibodies specific for epitopes on AMH_N_ (AMH-39/48) and AMH_C_ (AMH-39/29) were used separately.

### Various AMH Cleavage Products Were Detected in FFs and Associated GCs From the Same Woman

Various AMH cleavage products were detected in FFs and associated GCs using antibodies specific to AMH_N_ (i.e., AMH-39/48) and AMH_C_ (i.e., AMH-39/29) (**Figure 4C**). These data clearly illustrate that within one follicle, AMH forms differ between FF and GCs and that low molecular weight fragments of 14 and 16 kDa were only detected in GCs (**Figure 4C**, dotted box). **Figure 4D** displays the results from the same samples as in **Figure 4C**. Antibodies specific to AMH_N_ (i.e., AMH-39/48) and AMH_C_ (i.e., AMH-39/29) were used simultaneously under nonreduced and reduced conditions. Under reduced conditions, high-intensity bands at 55 kDa were detected in both FFs, GCs, and standards. The low molecular weight recombinant AMH standard was reduced from 20 to 12 kDa, whereas the low molecular weight bands in GCs at 16 and 14 kDa did not change molecular weights (**Figure 4D**, dotted boxes).

## Discussion

The present study demonstrates that the number of AMH isoforms in FF and GCs found in human small antral follicles are unexpectedly high, and highly variable between follicles and between FFs and corresponding GCs in a large set of samples. In addition, the distribution of AMH isoforms in GCs differs profoundly between follicles, even in follicles of the same size from the same woman. Furthermore, GCs express AMH-related peptides that, to our knowledge, have not previously been described. The ELISA data and western blot analysis show that the processing of AMH does not follow the patterns obtained with *in vitro*-produced recombinant AMH. The processing of AMH in the human follicular compartment appears to be more complex than previously described; several unknown cleavage products were detected.

In western blots, we detected bands at 125 kDa (proAMH homodimer), 110 kDa (AMH_N_ homodimer), 100 and 87 kDa (novel cleavage fragments), and 55 kDa (AMH_N_ monomer). Furthermore, bands at 16 and 14 kDa were detected in GCs, possibly representing AMH_C_ fragments since the staining is only seen using the antibody that recognizes AMH_C._ An increased rate of proAMH cleavage was detected in 4–9 mm follicles, giving rise to a strong 110 kDa band in these FFs, indicating that the smallest follicles contribute the most to bioactive AMH (**Figure 4A**). This finding fits with the observation that CYP19 remains downregulated in these small follicles and only becomes upregulated after follicle selection at a diameter of 8–10 mm (19). The AMH_C_ is normally ascribed a molecular weight of approximately 25 kDa, which under reducing conditions results in a molecular form of approximately 12 kDa. This is about the same as that observed in the present study using a recombinant AMH standard. Interestingly, a prominent 14- and 16-kDa band is detected in several of the GC samples using an antibody specific to AMH_C_, which furthermore is unaltered by exposure to reducing conditions (**Figure 4D**). This indicates that no disulfide bridges are present in these molecular forms, and they appear to consist of one peptide chain. It is surprising to note that different AMH isoforms present in GCs do not match those present in the corresponding FFs. The different Mw bands may be alternative cleavage products, different glycosylation forms or degradation products, caused by enzymatic catalysis in GCs and FFs (35). Alternatively, AMH_C_ may have an intracellular function as a transcription factor in GCs (33) and are to a lesser extent released in the FFs.

To our knowledge, this finding has not been reported in the literature and raises the question of whether these AMH-related peptides exert physiological functions. In this context, it is worth noting that we detected nuclear localization using immunohistochemical staining of cell nuclei in some follicles using the AMH_C_-detecting antibody (i.e., 39/29 antibody) (33), while the AMH-39/48 antibody showed no nuclear staining. This may suggest that autocrine regulation and processing of AMH within human GCs takes place, which warrants further investigation.

These patterns of AMH peptides, detected by the two antibodies used for western blotting, demonstrate that AMH is proteolytically processed in different ways and suggest additional cleavage sites. These results, in combination with the four ELISAs employed on human FF samples from small antral follicles (in which a the same AMH standard was used), demonstrate that it will be difficult to develop a single assay to detect all AMH-related peptides in a biological sample. Furthermore, it will be difficult to produce a standard that can be used to define an AMH concentration in each sample. Previous studies have evaluated the performance of commercial AMH assays in serum and found very different performances between assays. In some assays, AMH was hardly detected. Other assays found normal concentrations of AMH corresponding to the number of follicles present (36, 37), probably reflecting that the antibodies employed captured different epitopes. It is important to note that the concentration of AMH in FFs is a thousand times higher than it is in serum (38). Furthermore, some of the AMH isoforms present in FFs and GCs may be degraded in serum, explaining why we do not expect the isoform profiles detected in the follicular compartment to be reflected in serum. Most likely, the low-Mw AMH_C_ isoforms detected in GCs are unglycosylated and therefore rapidly cleared in circulation. It has previously been demonstrated that proAMH and AMH_N,C_ are the only confirmed isoforms present in serum, with proAMH being the dominant form (27). This suggests that assays designed for measurements in serum should preferably capture and detect at the N-terminal because this appears to be the most stable isoform. The present results suggest that a combination of two or more capturing antibodies or a single epitope two-sided antibody should be used to assure binding of as many AMH-related peptides as possible. However, a combination of antibodies targeting both the N- and C-terminal parts of AMH could potentially result in measurements of cleaved fragments and thereby cause overestimation.

The AMH_N_-specific antibody detected high molecular weight AMH bands of 140, 110, and 87 kDa in the recombinant AMH standard, which were similar to the bands detected in GCs but different from the bands detected in FFs (i.e., 100 and 55 kDa). The AMH_C_-specific antibody detected only one low molecular weight band in the recombinant standard of 23 kDa, whereas this antibody detected 16 and 14 kDa bands in GCs and no low molecular weight bands in FFs. Moreover, this AMH_C-_specific antibody detected an 87 kDa band in FFs, which was not present in GCs, suggesting that further proteolytic cleavage takes place in FFs that does not take place in the GCs.

Additionally, high molecular weight bands of 140, 110, and 100 kDa were detected with this AMH_C_-specific antibody in both FFs and GCs; the 110 kDa fragment could be an AMH_N_ homodimer, whereas the others may be alternative cleavage forms of AMH.

The four ELISAs, specific for a unique pair of epitopes on the AMH molecule, detected very different concentrations of AMH-related peptides. We found a significant negative correlation between the follicle diameter and the concentration of AMH isoforms measured with three of the four ELISAs, supporting previous findings (14, 19, 32). The assay capturing and detecting the AMH_C_ did not detect a significant correlation between follicle diameter and AMH concentration, suggesting that the contribution of AMH_C_ may be constant regardless of the size of the follicle. Since the AMH_C_ is not glycosylated, it is likely to be cleared from circulation relatively fast, which is corroborated by its near absence in circulation (27). Thus, it may be hypothesized that AMH exerts a biological function primarily within follicles, where the concentration is much higher than that in circulation, which on the flip side suggests that AMH may not be biologically active outside of the follicle in circulation and other biological fluids, where the concentration is much lower and AMH_C_ is not present. This further demonstrates that AMH in FFs is present in multiple molecular forms.

Our study demonstrates that AMH expression increases in cumulus cells surrounding the oocyte, showing a gradient with lower expression in mural cells and thereby variable AMH expression per cell. This result is similar to that observed in sheep follicles (8) and suggests that the oocyte affects AMH expression in cumulus cells. In a previous study, we found that AMH remained highly expressed in cumulus cells compared to mural GCs (39), which corroborates the results of this study. The functional significance of this finding is not revealed by the study. However, it can be hypothesized that the increased AMH expression in cumulus cells may inhibit aromatase expression and thereby the conversion of androgens to oestrogens, which has been shown in sheep follicles (8). Faint AMH staining was detected in theca cells, which may represent AMH bound to AMHR2 (40). A similar observation was made in human foetal male mesonephri and explained by AMH bound to its receptor (33).

The present study has taken advantage of the high concentrations of AMH in FFs and GCs to obtain a detailed picture of the various molecular isoforms in FFs that are present in human small antral follicles. The vast majority of AMH is produced within small antral follicles, from which AMH diffuses to circulation. Thus, it is conceivable that the molecular isoforms of AMH in follicles will resemble those in circulation. However, the individual follicles may show pronounced differences, which in circulation will be the average of what is released from the pool of ovarian follicles. In addition, further proteolytic processing may take place in circulation, giving rise to additional cleavage products that may not reflect peptides within the follicles.

Taken together, these results demonstrate the complexity of predicting the biological activity of AMH detected in FFs and GCs. AMH cleavage products differ between FFs and GCs from the same follicle and between follicles with the same diameter from the same women, illustrating that processing AMH gives rise to additional cleavage products, some of which may be active and some inactive. As ovarian GCs are the main producer of AMH in women, the molecular isoforms present in circulation are likely to reflect those in FFs and GCs; therefore, it is difficult to envision an ELISA that will detect the various AMH isoforms with similar efficacy in different serum samples. This suggests that serum AMH measurements and their clinical outcomes may depend on the isoform measured by a specific ELISA. In addition, it will be difficult to define an AMH measurement protocol that will standardize the content of AMH in different samples.

## Data Availability Statement

The original contributions presented in the study are included in the article/**Supplementary Material**. Further inquiries can be directed to the corresponding author.

## Ethics Statement

The studies involving human participants were reviewed and approved by The Scientific Ethical Committee for the Capital Region, Kongens vænge 2, 3400 Hillerød, Denmark. Written informed consent to participate in this study was provided by the participants’ legal guardian/next of kin.

## Author Contributions

LM wrote the paper, performed the statistical analysis, obtained the FFs and GCs in connection with ovarian cortex cryopreservation, assisted in WB, prepared figures and tables, and interpreted data. JJ and JB did the IF analysis. JB, SK, and SP obtained and processed FFs and GCs. AK and BK developed the antibodies and the ELISAs used (Ansh Labs, LLC, Webster, TX, USA) and interpreted the data. EE recruited patients for the project and performed the ovariectomies. CA interpreted the data, assisted in writing the paper, and was responsible for the study design. All authors critically revised the manuscript. All authors contributed to the article and approved the submitted version.

## Funding

Rigshospitalets Forskningspuljer, Novo Nordic Foundation, The Independent Research Fund Denmark, and the EU interregional project ReproUnion 2.0 funded this study but had no role in the study design, collection, and analysis of data, data interpretation, or writing. The corresponding author had full access to all data presented in the study and made decision to submit this article for publication.

## Conflict of Interest

Authors AK and BK were employed by the company Ansh Labs.

The remaining authors declare that the research was conducted in the absence of any commercial or financial relationships that could be construed as a potential conflict of interest.
