# Supplementary material for: High Variability of Molecular Isoforms of AMH in Follicular Fluid and Granulosa Cells From Human Small Antral Follicles
Source: Front Endocrinol (Lausanne). 2021 Mar 2;12:617523. doi: 10.3389/fendo.2021.617523 (PMC7961079; doi:10.3389/fendo.2021.617523)
Supplement: Supplementary file 1 [file Table_1.pdf]

| Sample no. | Follicle diameter (mm) | 24/32 (ng/mL) | 24/37 (ng/mL) | 32/33 (ng/mL) | 10/24 (ng/mL) |
|---|---|---|---|---|---|
| 1 | 4 | 1672.1 | 1102.7 | 198.1 | 807.6 |
| 2 | 4 | 1212.2 | 1227.9 | 188.3 | 937.4 |
| 3 | 4 | 111.4 | 66.2 | 26 | 63.7 |
| 4 | 4 | 1120.2 | 631.2 | 79.9 | 509.3 |
| 5 | 4 | 130.6 | 142.2 | 22.2 | 109.8 |
| 6 | 5 | 1467.5 | 918.7 | 180.7 | 697.1 |
| 7 | 5 | 473.7 | 443.1 | 197.3 | 388.7 |
| 8 | 5 | 1247.2 | 719 | 52.9 | 650.9 |
| 9 | 5 | 1035.2 | 508 | 119.7 | 462.9 |
| 10 | 5 | 779.6 | 510.4 | 85.9 | 399.5 |
| 11 | 5 | 8.3 | N/A | N/A | N/A |
| 12 | 5 | 234.6 | 235 | 30.4 | 187.7 |
| 13 | 5 | 153.2 | 150.9 | 28.9 | 128 |
| 14 | 5 | 1045.7 | 629.6 | 128.7 | 503.4 |
| 15 | 5 | 1616.5 | 966.4 | 205.7 | 825.1 |
| 16 | 6 | 1600 | 956.9 | 189 | 805.2 |
| 17 | 6 | 286.3 | 265.4 | 48.4 | 230.6 |
| 18 | 6 | 50.9 | 41.3 | 23 | 29.1 |
| 19 | 6 | 1256.9 | 814.4 | 246 | 674.7 |
| 20 | 6 | 1803 | 1040.3 | 293.4 | 876.4 |
| 21 | 6 | 133.6 | 91.5 | 32.7 | 85 |
| 22 | 6 | 888 | 584.4 | 39.4 | 423.5 |
| 23 | 6 | 303.5 | 182.9 | 40.9 | 148.4 |
| 24 | 6 | 1729.4 | 1103.6 | 238.3 | 880.5 |
| 25 | 6 | 138.9 | 107.4 | 12.7 | 78.7 |
| 26 | 6 | 1066.7 | 550.8 | 225.4 | 497.5 |
| 27 | 7 | 261.9 | 180.7 | 10.5 | 136.5 |
| 28 | 7 | 303.5 | 207.1 | 15.6 | 159.6 |
| 29 | 7 | 570.5 | 360.1 | 79.1 | 282.8 |
| 30 | 7 | 1532.9 | 957.7 | 180.7 | 808.4 |
| 31 | 8 | 209.9 | 142.2 | 16.3 | 101.3 |
| 32 | 8 | 1206.1 | 708.2 | 187.5 | 587.1 |
| 33 | 8 | 934.4 | 597.3 | 150.5 | 467.3 |
| 34 | 8 | 480.5 | 321.5 | 92.6 | 243.2 |
| 35 | 8 | 1079 | 662.2 | 177.7 | 539.8 |
| 36 | 9 | 72.4 | 50.9 | 11.2 | 40.7 |
| 37 | 9 | 1272.6 | 803.4 | 128.7 | 618.9 |
| 38 | 9 | 449.9 | 260.2 | 92.6 | 225.6 |
| 39 | 10 | 31.2 | 16.7 | 5.6 | 10.9 |
| 40 | 10 | 118 | 95.9 | 8.4 | 60.8 |
| 41 | 10 | 5.6 | N/A | N/A | N/A |
| 42 | 10 | 71 | 49.4 | N/A | 41.5 |
| 43 | 10 | 121 | 94.4 | 19.3 | 74.4 |
| 44 | 11 | 27.6 | 13.6 | 5.6 | 8.7 |
| **Mean** | | **689.0** | **465.0** | **100.4** | **376.4** |
| **Min** | | **5.6** | **13.6** | **5.6** | **8.7** |
| **Max** | | **1803.0** | **1227.9** | **293.4** | **937.4** |
| **SD** | | **591.6** | **366.1** | **84.6** | **295.8** |
